# Update from New Zealand

**DOI:** 10.1186/1471-2393-15-S1-A3

**Published:** 2015-04-15

**Authors:** Edwin A Mitchell

**Affiliations:** 1 Department of Paediatrics, University of Auckland, Auckland, New Zealand

## 

The Auckland Stillbirth Study, a prospective population-based case control study, was the first study to report maternal sleep related practices as risk factors for stillbirth [1]. In brief there were 155 women with a singleton late stillbirth (at or greater than 28 weeks’ gestation) without congenital abnormality, born between July 2006 and June 2009 and booked to deliver in Auckland. They were compared with 310 women with single ongoing pregnancies and gestation matched to that at which the stillbirth occurred. The prevalence of late stillbirth in this study was 3.09/1000 births. No relationship was found between snoring or day time sleepiness and risk of late stillbirth. However, women who slept on their back or on their right side on the last night (prior to stillbirth or interview) were more likely to experience a late stillbirth compared to women who slept on their left side (back: aOR 2.54; 95% CI: 1.04 to 6.18; and right side: aOR 1.74; 95% CI: 0.98 to 3.01). Women who got up to the toilet once or less on the last night were more likely to experience a late stillbirth compared to women who got up more frequently (aOR 2.28; 95% CI: 1.40 to 3.71). Women who regularly slept during the day, in the last month, were also more likely to experience a late stillbirth compared to those who did not (aOR 2.03; 95% CI 1.26 to 3.27). If maternal sleeping position is causally related to stillbirth then 37% of stillbirths might be prevented if mothers slept on their left side. However, the authors urged caution as this was the first time that an association has been described between maternal sleep practices and late stillbirth risk. Further studies are needed to confirm or refute these findings before public health interventions are launched.

In New Zealand there is a multicentre case-control study, which began in 2012, led by Lesley McCowan, and in England there is the Midland and North of England Stillbirth Study (MiNESS), which began in 2014, led by Alex Heazell [2].

Despite urging caution, midwives appear to have accepted the findings and are advising their patients to sleep on the left side. This has resulted in a significant increase in left sided sleep position, from 35.9% in The Auckland Stillbirth Study (2006-9) to 62.5% in late 2011 (unpublished findings). This has been associated with a reduction in late stillbirth for New Zealand (excluding congenital abnormalities and multiple pregnancies).

2007 184

2008 187

2009 205

2010 162

2011 146

2012 136

Although we cannot exclude other reasons for the decline, it is tempting to believe that the declined is a consequence of more pregnant women sleeping on their left.
